# Researches on Hydrides and Hydrates

**Published:** 1894-05-26

**Authors:** Benjamin Ward Richardson


### RESEARCHES ON HYDRIDES AND HYDRATES.

By Sir BENJAMIN WARD RICHARDSON, M.D., F.R.S.

I ONCE made a careful study, for therapeutical purposes, of certain of the chemical bodies known as hydrides and hydrates, and as some of these have come of late years into use for medicinal purposes, I will devote a little attention to them in these pages, reporting most freely on those which have received least attention.

The hydrides I studied were four in number, methyl, ethyl, butyl, and amyl hydride. The first is sometimes called protylen, the second deutylen, the third tetrylen, and the fourth pentylen. The first three are gases, the last is a very light fluid. They are in every case compounds of hydrogen, combined with the respective radicals, methyl, ethyl, butyl, and amyl, and increasing in weight by addition of the carbon equivalent. Methyl hydride is composed of $C\,H_3\,H$; ethyl hydride of $C_2\,H_5\,H$; butyl hydride of $C_4\,H_9\,H$; and amyl hydride of $C_5\,H_{11}\,H$. The three first are gases, having vapour densities respectively of 8, 15, and 29, taking hydrogen 2=1. The amyl compound, as already stated, is a light fluid, with a vapour density of 36 and a specific gravity of ·625, taking water as 1,000. It boils at 30 deg. C. or 86 deg. Fahr.

### HYDRIDE OF METHYL OR PROTYLEN.

This substance is commonly known as fire-damp in mines, and as marsh gas, on land. It is easily made by heating together acetate of soda, caustic potassa, and well dried lime. It is a very explosive gas, but is inodorous to breathe, and, in its physiological action, is simply anæsthetic. In order that it may produce quick anæsthesia, it must, like nitrous oxide, be undiluted with air. If it be much diluted it produces an ethereal excitement before causing deep sleep. Its light vapour density leads it rapidly to escape in expiration, and this the more easily owing to its great insolubility in blood. The late Mr. Nunnely, of Leeds, first thought of using this gas for anæsthesia, and introduced it as an anæsthetic in the year 1849. It underwent no further research until my own work with it in 1886, reported on in 1867.

If the gas be inhaled diluted with air, so that the air breathed is charged with 35 per cent. of it, slow anæsthesia can be induced in warm-blooded animals, but a lesser per cent. does not influence until after a long period. If, however, it be inhaled as the pure gas, well washed, at a temperature of 60 deg. to 70 deg. Fahr., anæsthesia is extremely rapid in its development, and that without excitement, and without the slightest irritation of the larynx, tremor, or convulsive spasm. In my first report upon its action I suggested that it produced its effects by inducing a process of gradual negation of respiration, that is to say, by replacing the air required for the oxidation of blood, much as if it were nitrogen or other perfectly negative gas. I am still inclined to that opinion.

It is as yet uncertain whether methyl hydride could be used safely as an anæsthetic in place of nitrous oxide for short operations, such as extraction of a tooth. I administered it to the late Dr. Clarke, of Henrietta Street, W., while Mr. Peter Matthews extracted a firm molar. I also administered it in six similar operations, by the same dentist, at the Marylebone Dispensary. In all cases it acted well, sufficient insensibility being induced to enable the operator to extract without pain in from twenty to thirty seconds, the gas being entirely undiluted with air. Recovery in every case was perfect within a minute after the extraction, and I saw no untoward symptom whatever, except that Dr. Clarke, who was a man advanced in years, complained for a few minutes of a slight giddiness after he returned to consciousness. The great difficulty I experienced was in the administration of a gas that had to be used in very large quantities, and to be retained over water in a cumbrous receiver. In these days when compression of gases is comparatively easy, that difficulty might, perhaps, be overcome in the same way as with nitrous oxide, carbonic acid, oxygen, and some other gases.

A point of physiological interest is connected with this gas in that it is a gas in which miners sometimes lose their lives. It has been observed that the dead men, in these instances, have been found in the most placid form, showing no sign of struggle or convulsion, and retaining a natural expression of face as regards both complexion and feature. I have no doubt, from what I have observed, that the lethal state brought on by the inhalation of the gas is perfectly painless, and that death could not be inflicted in a more easy and unconscious manner. I should think it is almost impossible for men in a mine to be so entrapped with fire-damp while they were at work as suddenly to die in the absence of an explosion; but after an explosion, when the mine becomes, for the moment, a great vacuum, there might be sufficient entrance of gas to produce a fatal atmosphere.

In animals killed by the gas—that is to say, animals allowed to sleep to death in it—I found the lungs always charged with blood, the heart itself charged with blood on both sides, and the blood of natural colour, arterial and venous. It was observed, however, that the irritability of the heart was not long maintained.

One further fact deserves to be noted. From the circumstance that the hydride exists in the air of marshes it has been conceived as a cause of malarial fever. I see no reason to believe that it has any such action, and must suppose that the true poison at work in malarial fever is some different agent altogether, which may be present, coincidently, with the gas. There was nothing in my observation that conveyed the slightest suspicion of methyl hydride having any other action than that of causing anæsthesia.

It was in connection with experiments with this gas —methyl hydride—that I made the attempt to construct what I called the miner's mask, namely, a mask which, worn by a miner when he was in danger from inhalation of the gas, would exclude the lethal agent, and allow the common air to be respired. I desisted on being assured that success would be failure, since no miner would adopt the use of the mask; but the research, as I may one day show, was not without its value.

